# Imaging: New Frontiers in Vascular Training

**DOI:** 10.14797/mdcvj.1093

**Published:** 2022-06-03

**Authors:** Kavya Sinha, Marton Berczeli, Alan B. Lumsden, Trisha L. Roy

**Affiliations:** 1 Houston Methodist Hospital, Houston, Texas, US; 2 Semmelweis University, Budapest, Hungary; 3 Houston Methodist DeBakey Heart & Vascular Center, Houston Methodist Hospital, Houston, Texas, US

**Keywords:** vascular imaging, vascular surgery, medical education, fellowship

## Abstract

Advances in medical imaging have redefined the practice of vascular surgery. Current training programs for vascular surgery do not incorporate formal training in vascular imaging other than in duplex ultrasound when a physician is undergoing the vascular interpretation certification process. Yet imaging modalities and techniques have grown exponentially in the adjacent fields of interventional radiology, interventional and diagnostic cardiology, and neuroradiology, so much so that advanced imaging fellowships have been established in these fields. This article reviews the current state of vascular imaging training, identifies gaps in the current training regimen, and proposes an advanced vascular imaging fellowship for the future.

## Introduction

Advances in medical imaging have redefined the practice of vascular surgery. The trend towards minimally invasive procedures, which rely heavily on guidance from imaging modalities, has improved patients’ recovery times and outcomes. However, no formal training is available in multimodal vascular imaging and the advances that exist.^1^ Digital subtraction angiography, computed tomography angiography, and duplex ultrasound remain the most commonly used imaging methods in vascular surgery, but these techniques have had limited advancement in teaching and training in the past 20 years. Duplex ultrasound now has a formalized curriculum through the Registered Physician in Vascular Interpretation certification process, which is required to become board certified in vascular surgery. However, vascular surgeons have no role in the interpretation of other imaging modalities, and many feel uncomfortable interpreting magnetic resonance imaging (MRI), which has ultimately limited its clinical use due to the limited training vascular surgeons receive in MRI. The inability to understand, post-process, and interpret images may inhibit the growth of the vascular surgery specialty if surgeons feel unable to keep up with state-of-the-art imaging techniques that deliver the best possible care.^1^ The purpose of this review is to discuss the current state of training in vascular imaging, identify gaps in knowledge and training, and propose an advanced vascular imaging fellowship for the future.

## The Transition from Vascular Surgeon to Vascular Specialist

**Figure 1:**
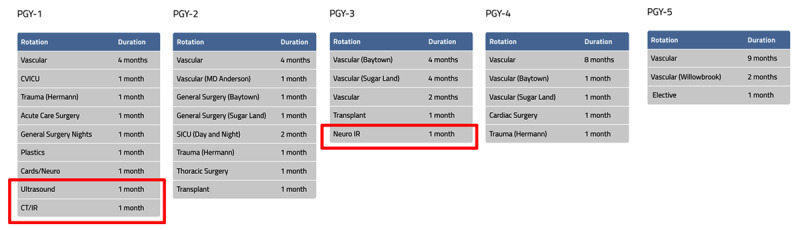
Integrated 0+5 vascular surgery residency curriculum; red boxes indicate training rotations related to imaging.

In 2006, the Accreditation Council for Graduate Medical Education (ACGME) started the first integrated 0+5 vascular surgery residency programs to address the changing scope of vascular surgery and rebrand the specialty to encompass the holistic care of vascular patients (***Figure 1***). The goal was to train the next generation of vascular specialists to become experts in vascular diagnostics, medical therapies, endovascular training, and open surgery.^2,3,4,5^ This change in the training paradigm recognized the fact that general and vascular surgery skill sets diverged with the increased use of laparoscopic and fluoroscopic techniques, respectively. However, it is challenging to understand the vascular specialist’s role and differentiate it from specialties such as interventional cardiology and interventional radiology. Traditional training often invokes “The Law of the Instrument,” arguing that “if your only tool is a hammer, everything looks like a nail”; in this context, vascular specialists have the unique ability to personalize treatment for patients in order to offer both open and endovascular treatments. At the same time, this argument fails to address the fact that vascular surgeons have limited their “tools” by not keeping pace with the multimodal imaging that dictates most vascular care today.

The ability to understand and interpret imaging is critical to treating patients with vascular disease. Cardiology has shown how dedicated training in advanced imaging has had the synergistic effect of giving cardiologists more information to treat patients while also improving the imaging modalities themselves.^4,5^ As an “end-user” of the imaging techniques, the perspectives of cardiologists combined with imaging training have led to major advances in cardiac MRI, coronary computed tomography (CT), positron emission tomography perfusion imaging, and more. Improved diagnostics propelled the cardiology specialty as a whole as clinicians developed a new understanding of the structure and function of the heart and vessels. By comparison, vascular surgery has not seen similar growth in imaging techniques, and the lack of training has limited the specialty as a whole from integrating and developing new imaging modalities into clinical practice.^5^

## Current Imaging Training for Vascular Specialists

While some degree of variability exists in the curriculum for integrated 0+5 vascular surgery residencies across the country, dedicated imaging training is not typically a focus (***Figure 1***).^1^ For example, in the curriculum for the 5-year integrated vascular surgery residency training program at Houston Methodist Hospital, first-year residents have 1 month of CT or interventional radiology training and 1 month of duplex ultrasound training. They also have a 1-month interventional neurology rotation in the third year. These 1-month rotations help residents gain exposure to more types of cases and disciplines. However, a month is typically not enough time to gain the necessary understanding and knowledge to accurately interpret multimodal imaging modalities. Apart from these dedicated 3 months, residents learn to interpret images throughout their training in case conferences and day-to-day clinical activities that require a cursory knowledge of CT angiography and x-ray angiography. Ultrasound education is the exception, because the Registered Physician in Vascular Interpretation qualification, which requires ultrasound training, is a prerequisite for vascular surgery certification. Ultrasound interpretation has become a key competency in vascular surgery and highlights the value of gaining the necessary training to interpret vascular imaging.^4,5,6^

## Vascular Imaging Gaps in Knowledge

Significant gaps in knowledge with respect to vascular imaging are not addressed in the current training paradigm. This section provides examples of such gaps as well as future opportunities for patient selection, procedure planning, intraoperative guidance, and long-term patient surveying.

### Patient Selection

**Figure 2:**
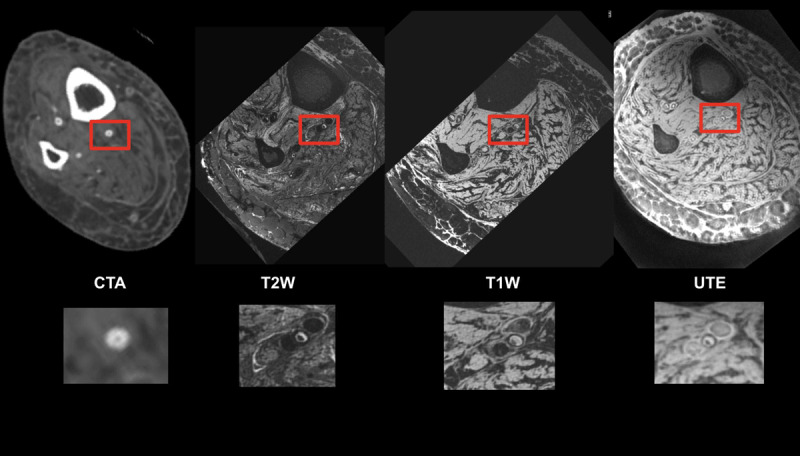
Tibial vessels imaged with computed tomography angiography (CTA) and magnetic resonance imaging (MRI) using T2-weighted, T1-weighted, and ultrashort echo time sequences. The CTA shows concentric calcium but suffers from beam hardening artifacts and calcium blooming that obscures the vessel lumen. MRI with ultrashort echo time imaging shows a very thin rim of hypointensity around the central vessel, indicating that the calcium ring is thin and the lumen is occluded with mixed morphology plaque.^11^

Appropriate patient selection is the cornerstone of optimal vascular care. Patient age, comorbidities, and clinical exam inform management, but the final decision of whether to intervene either endovascularly or via traditional open surgery hinges on imaging. Typically noninvasive vascular lab studies are reviewed first, followed by cross-sectional imaging with CT angiography and at times intraprocedural x-ray angiography.^7,8^ Peripheral artery disease is growing exponentially, but the current imaging modalities have major limitations, particularly for tibial imaging. X-ray angiography is still considered the gold standard imaging technique, but it is invasive, user-dependent, requires significant amounts of nephrotoxic contrast, and exposes the patient and operator to radiation.^7^ Most importantly, x-ray angiography is not as sensitive for tibial images or for patent vessels that are missed, which could be visualized with MR angiography.^7^ Many vascular surgeons are not confident in interpreting vascular MRI and avoid using it in daily practice. However, it is a powerful imaging modality capable of plaque characterization, flow-independent angiography, hemodynamics, and perfusion assessments. It is also a safe technique that is noninvasive and does not require contrast.^8,9,10^ ***Figure 2*** provides a comparison between CT angiography and MRI.^11^ With more training and familiarity, vascular surgeons can augment their practice by using the full breadth of MRI techniques. Training also enables vascular surgeons to participate in the development of novel MRI methods similar to the impact that cardiologists had in developing cardiac MRI (CMR) techniques. CMR has changed the standard of care for cardiology patients and is now considered the gold standard for morphologic assessment of the heart and ventricular function.^4^

### Image Post-Processing for Preoperative Planning

Most vascular surgeons use standard clinical imaging modalities to plan procedures. However, the captured data contains a wealth of knowledge that benefits from awareness of post-processing to aid procedural planning. Most vascular surgeons have some degree of familiarity with sizing an endograft using centerline software such as TeraRecon (TeraRecon Inc.) or syngo.via (Siemens Healthineers).^11,12^

**Figure 3:**
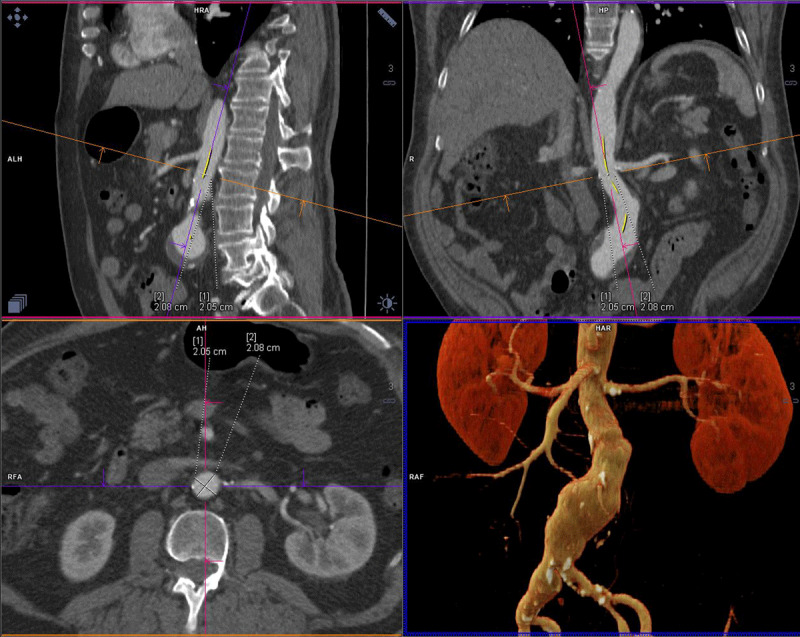
Three-dimensional multiplanar reconstruction determines aortic neck diameter, angle, and centerline measurements to size abdominal aortic endograft.

**Figure 4:**
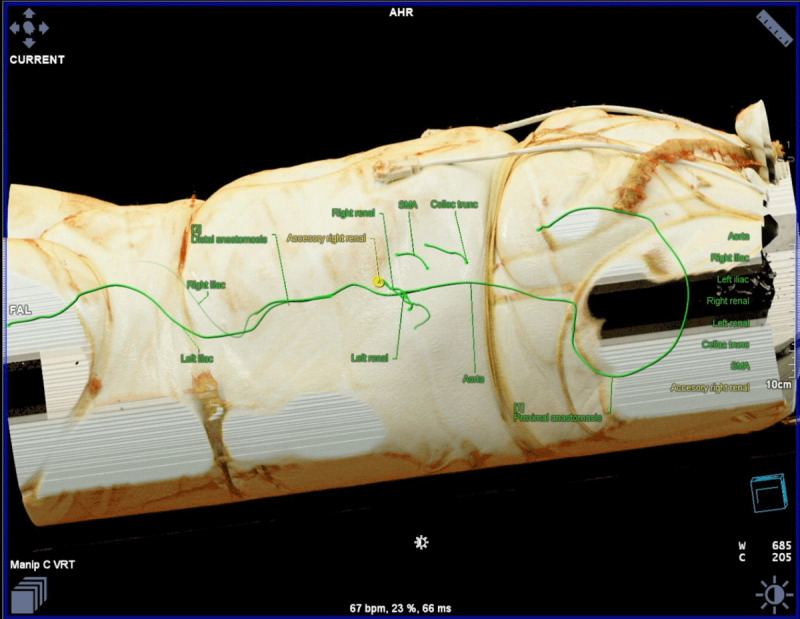
Cinematic rendering helps plan the optimal incision and approach for open repair of type 4 thoracoabdominal aneurysm.^11,13^

Although not used routinely, three-dimensional (3D) image post-processing software for planning open surgical procedures could offer important information. For example, “cinematic rendering” is a visualization tool that enables photo-realistic 3D visualization of vessels and surrounding tissue. Developed by Siemens Healthineers and still only approved for research purposes, this tool illustrates the potential for improving open surgical planning. This technique allows the user to scroll back and forth through layers of the body and from skin to muscle/soft tissue to visceral structures and bones. This enables surgeons to effectively plan their procedure and mentally rehearse the steps of complex exposures. ***Figure 3*** shows the cinematic rendering of a patient prior to open repair of a thoracoabdominal aortic aneurysm. This post-processing technique allows surgeons to select the optimal intercostal to enter the chest cavity and also provides information about the angles and locations of vital branch vessels that require individual bypass grafts or reimplantation (***Figure 4***).^11,13^ Though patient position affects the accuracy of the rendered images, surgeons nevertheless gain pertinent information to help plan open operations and optimize their approach.

### Intraoperative Procedural Guidance

Multimodal intraoperative imaging is playing a larger role in vascular procedures. In addition to using x-ray angiography, vascular surgeons typically use duplex ultrasound for arterial access and completion studies after carotid or bypass surgery. Use of intravascular imaging during vascular procedures also is increasing. Intravascular ultrasound (IVUS) is now the standard of care when intervening on aortic dissections to differentiate true and false lumens and is recognized as the gold standard imaging modality for venous interventions. Understanding IVUS and interpretation is taught informally during procedures, but other intravascular modalities such as optical coherence tomography (OCT) typically are not used.^14^ Even x-ray angiography, which is a routine modality used in vascular surgery, has specific training gaps. For example, there is no formalized curriculum for radiation safety, and practices vary significantly in terms of adherence to “as low as reasonably achieved” (ALARA) guiding principles.^5,15^ One way of reducing radiation for complex procedures is to use intraoperative image fusion with preoperative cross-sectional imaging using 2D or 3D image registration for guidance. Typically, this process involves registering preoperative CT angiography with intraoperative x-ray angiography, and its use in vascular surgery is limited primarily to large vessels of the aorta and major branches. However, interventional neuroradiology and interventional radiology routinely use CT guidance or rotational angiography with registration for small vessels, and some centers use time-resolved CT angiograms (4D CTA) in the operating room.^16^ By training with these other specialties, vascular surgeons can expand their toolbox to perform procedures precisely under image guidance. Registration and intraprocedural image guidance are likely to be safer for patients by reducing radiation, which is ultimately safer for the operating physician as well.

### Surveillance and Follow-Up Imaging

**Figure 5:**
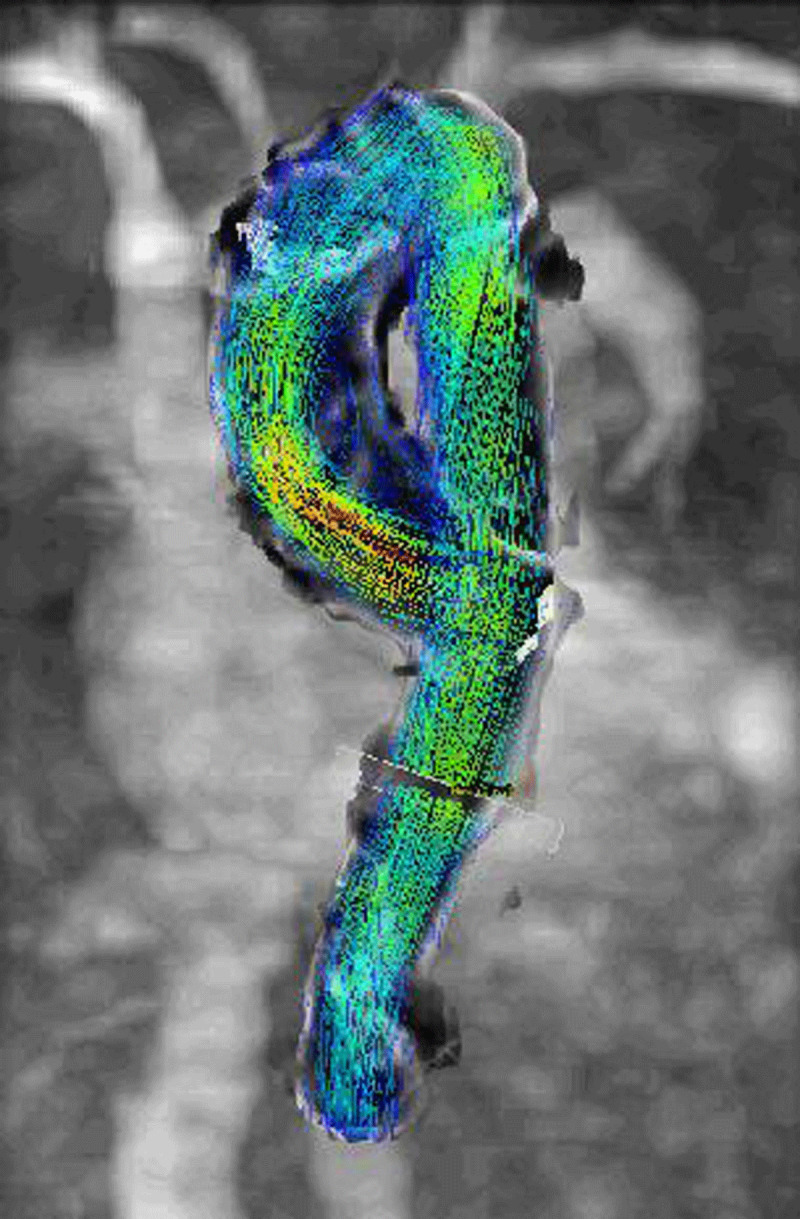
An example of four-dimensional (4D) flow magnetic resonance imaging, which enables the visualization of the temporal evolution of complex blood flow patterns within an acquired 3D volume. The blood flow patterns are represented in the colored vectors, which represent velocity with blue being closer to 0 m/s and red up to 1 m/s.^17,18,19^

Surveillance with duplex ultrasound is an established method for evaluating many conditions. Pertinent examples include surveying peripheral bypass grafts and endovascular repairs for abdominal aortic aneurysms at regular intervals.^8,12,17^ The Society for Vascular Surgery has established specific duplex ultrasound criteria to indicate when to intervene prophylactically. However, thoracic aortic disease cannot be imaged by ultrasound and requires CT angiography for surveillance on a lifelong basis. This poses risks of additional radiation and potential renal impairment with repeated studies over a lifetime. Furthermore, there are no universally accepted anatomic criteria for the management of uncomplicated type B dissections or when to intervene. An estimated 20% to 55% of medically managed type B dissections will develop aneurysmal degeneration at 5 years, but there are no established imaging findings indicating who is most at risk.^8^ The risk of aneurysmal degeneration is related to the pressure and flow within the true and false lumen and fenestrations between, but these dynamic features are not captured on traditional CT angiograms. Four-dimensional flow with MRI enables the visualization and quantification of complex flow patterns (***Figure 5***), and this modality, in particular, is showing promise in evaluating predictive factors to determine who would benefit most from prophylactic thoracic endovascular repair.^17,18,19^ False lumen pressures and the identification and hemodynamic measurements of small flap fenestrations may prove useful in patient selection as well as optimal placement of thoracic endografts in the future.^12,20^ Therefore, select CMR fellowship programs are needed to provide training on how to interpret these images, the basics of image post-processing, and research opportunities to improve these techniques in the future.

## The Learning Curve for Vascular Imaging

The path we propose to expand learning opportunities is similar to advanced imaging fellowships in cardiology. These 1-to-2-year programs focus on cardiac MRI, CT, and echocardiography.^2^ Some of the imaging fellowships available are research focused, supporting academic advancement that also may further motivate individuals, especially with the growing need for publications required to gain academic appointment.^3^ Research-based specialization and courses in a specific modality are the current practices for specializing in vascular imaging. Much like the American College of Cardiology (ACC) establishing a consensus statement emanating from the Core Cardiology Training Symposium (COCATS) to standardize cardiology training, we recommend that the vascular surgery board and professional organizations such as the Society for Vascular Surgery and the European Society for Vascular Surgery establish a similar statement to standardize advanced vascular imaging training.^4^ According to the 2021 ACC advanced training statement on vascular medicine, Level III training pertains to advanced knowledge in diagnostic and therapeutic modalities for evaluating and managing vascular disease that enables one to direct a vascular laboratory, train others, and conduct advanced research in vascular medicine.^5^ However, the COCATS 4 Task Force 9 report did not provide detailed guidelines for Level III training in vascular medicine; rather, it described it in broad terms to provide context for trainees and clarify that these advanced competencies are not covered during the cardiovascular fellowship.^19,21^

**Figure 6:**
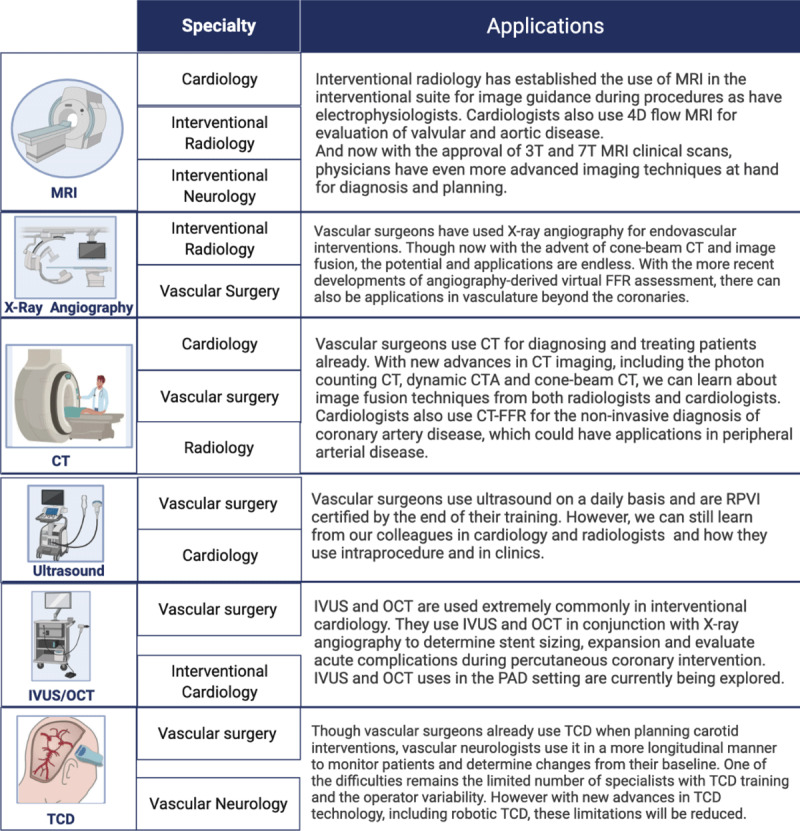
The strengths and capabilities of an interdisciplinary team in an advanced vascular imaging fellowship demonstrate the various applications of the different imaging modalities.^22,23^ MRI: magnetic resonance imaging; CT: computed tomography; IVUS/OCT: intravascular ultrasound/optical coherence tomography; FFR: fractional flow reserve; CTA: CT angiography; RPVI: Registered Physician in Vascular Interpretation; PAD: peripheral artery disease; TCD: transcranial doppler

Given the increasing burden of vascular diseases, it is critical that trainees in cardiovascular medicine receive advanced training in vascular medicine.^5^ By instituting an advanced imaging fellowship, we could train future vascular specialists to lead and direct labs with the complete spectrum of imaging modalities. However, the current limitation to establishing a vascular imaging fellowship is the lack of vascular surgeons with expertise in using imaging modalities such as CT or MRI, especially compared with the fields of cardiology or interventional radiology. Therefore, to overcome such limitations, we propose a team that includes faculty members from other disciplines such as cardiology, radiology, and neurology. An interdisciplinary team would enable the next generation of vascular specialists to read and interpret the various imaging modalities that would be applicable in both academic centers and private practice clinics (***Figure 6***).^22,23^

Furthermore, an interdisciplinary team would allow the different specialists to demonstrate how to read, interpret, and post-process the relevant modalities. Cone-beam CT training could be done by vascular surgeons and interventional radiologists, and interventional cardiologists could provide IVUS/OCT training. Transcranial Doppler training could be provided by vascular surgeons and vascular neurologists, whereas training in CT and MRI reading and interpretation could be done by radiologists and cardiologists. Interventional cardiologists have proven the utility of noninvasive diagnostic imaging such as CT fractional flow reserve for evaluating coronary artery disease, and image fusion with cone-beam CT and fluoroscopy is being used for intraprocedural guidance. Interventional radiologists use MRI and ultrasound fusion for targeted biopsies. By harnessing the experience of different specialties, advanced vascular imaging fellows can apply these techniques to their own practice. In addition, an interdisciplinary approach would train fellows to think outside the box and optimize imaging to improve delivery of care.

## Conclusion

**Figure 7:**
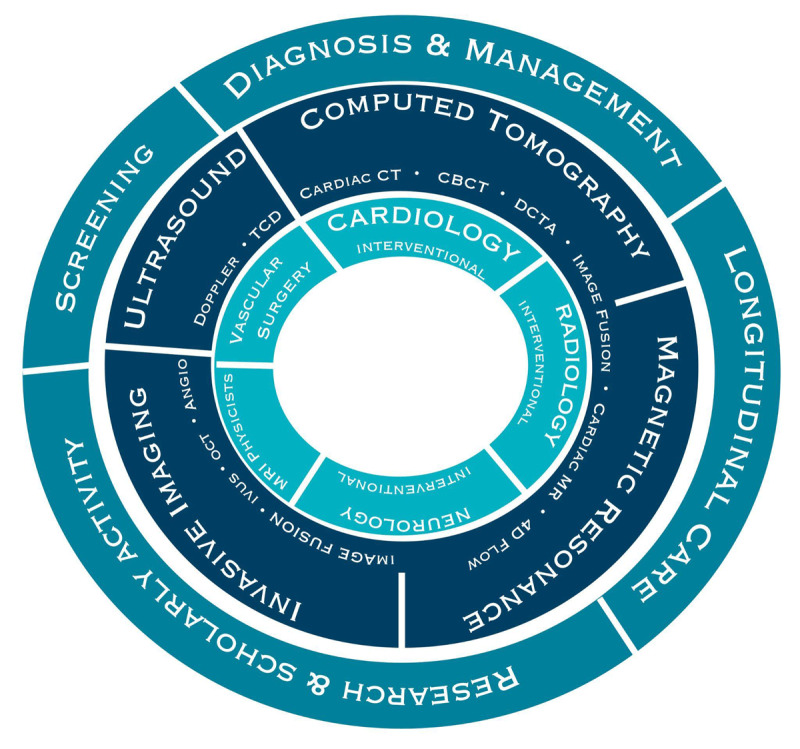
The core competencies of our proposed advanced vascular imaging fellowship. TCD: transcranial doppler; CBCT: cone-beam computed tomography; DCTA: dynamic CT angiography; IVUS: intravascular ultrasound; OCT: optical coherence tomography

By working with and learning from different specialists, vascular trainees can learn to apply the same techniques to their patients and continue the vascular surgery paradigm of personalized treatment plans. Our proposed advanced vascular imaging fellowship could equip fellows with all the core competencies required to screen, diagnose, and care for vascular patients while also enabling them to train future generations of vascular specialists. Similar to the COCATS diagram, ***Figure 7*** illustrates the importance of an interdisciplinary team and the collaboration needed to train vascular specialists who are knowledgeable in all applicable imaging modalities.

## Key Points

An advanced vascular imaging fellowship could enable fellows to be trained with all the core competencies required to:

- screen, diagnose, and care for vascular patients;
- learn the different imaging techniques and apply them in novel ways to treat patients; and
- apply a team-based approach in treating patients.
